# Antiperovskite Chalco-Halides Ba_3_(FeS_4_)Cl, Ba_3_(FeS_4_)Br, and Ba_3_(FeSe_4_)Br with Spin Super-Super Exchange

**DOI:** 10.1038/srep15910

**Published:** 2015-11-03

**Authors:** Xian Zhang, Kai Liu, Jian-Qiao He, Hui Wu, Qing-Zhen Huang, Jian-Hua Lin, Zhong-Yi Lu, Fu-Qiang Huang

**Affiliations:** 1 Beijing National Laboratory for Molecular Sciences and State Key Laboratory of Rare Earth Materials Chemistry and Applications, College of Chemistry and Molecular Engineering, Peking University, Beijing 100871, China; 2 Beijing Key Laboratory of Opto-electronic Functional Materials & Micro-nano Devices, Department of Physics, Renmin University of China, Beijing 100872, China; 3 CAS Key Laboratory of Materials for Energy Conversion and State Key Laboratory of High Performance Ceramics and Superfine Microstructure, Shanghai Institute of Ceramics, Chinese Academy of Sciences, Shanghai 200050, China; 4 NIST Center for Neutron Research, National Institute of Standards and Technology, Gaithersburg, MD 20899-6102, USA

## Abstract

Perovskite-related materials have received increasing attention for their broad applications in photovoltaic solar cells and information technology due to their unique electrical and magnetic properties. Here we report three new antiperovskite chalco-halides: Ba_3_(FeS_4_)Cl, Ba_3_(FeS_4_)Br, and Ba_3_(FeSe_4_)Br. All of them were found to be good solar light absorbers. Remarkably, although the shortest Fe-Fe distance exceeds 6 Å, an unexpected anti-ferromagnetic phase transition near 100 K was observed in their magnetic susceptibility measurement. The corresponding complex magnetic structures were resolved by neutron diffraction experiments as well as investigated by first-principles electronic structure calculations. The spin-spin coupling between two neighboring Fe atoms along the *b* axis, which is realized by the Fe-S···S-Fe super-super exchange mechanism, was found to be responsible for this magnetic phase transition.

Perovskite oxides of the general formula of *AB*O_3_ (*A* = alkali metal ions, alkaline metal ions, rare earth ions *etc*; *B* = transition metal ions; O = oxygen), are a large material family with abundant physical properties. Important physical effects of perovskite oxides include ferroelectrics (PbTiO_3_)^1^,^2^,^3^, colossal magnetoresistance (CMR, La_1–*x*_Ca_*x*_MnO_3_)^4^,^5^,^6^, dynamic random access memory (D-RAM, BaTi_1–*x*_Zr_*x*_O_3_)^7^,^8^, high temperature superconductivity (cuprate perovskites and derivatives)^9^,^10^,^11^,^12^,^13^, and so on. Surprisingly, perovskite halides *ABX*_3_ (*A* = Cs or methylamine; *B* = Sn, Pb; *X* = Cl, Br, I)^14^,^15^,^16^,^17^,^18^,^19^,^20^,^21^,^22^ have become popular in less than five years due to their very high solar conversion efficiency of up to >20%^23^. New perovskite materials have been infused with various undiscovered new physical functions.

Antiperovskites have similar structure to that of the perovskites, where the positions of the cation and anion constituents are reversed^24^,^25^. Different from the perovskite materials common in nature, the number of antiperovskites is much smaller. Most naturally occurring antiperovskite minerals were formed due to geological activities^24^. There are only several structure types of artificial antiperovskites so far. The metallic antiperovskites *M*_3_*AB* (*M* = Mn, Ni, Fe; *A* = Ga, Cu, Sn, Zn; *B* = N, C, B)^25^ with strong correlations among lattice, spin, and charge possess to cause such unusual physical properties as superconductivity (MgCNi_3_)^26^, negative/zero thermal expansion^27^, magnetostriction^28^, piezomagnetic^29^,^30^, and novel magnetoelectronic effects^31^. Recently, lithium rich antiperovskites (Li_3_O*X*, *X* = Cl, Br) are recognized as a new type of antiperovskites, which have demonstrated superionic conductivity of lithium ions in solid state batteries^32^,^33^,^34^.

As mentioned above, the perovskite halides *MA*Pb*X*_3_ (*MA* = methylamine) have successfully been used in solar cells^14^,^15^,^16^,^17^,^18^,^19^. It is interesting to investigate what properties will appear in the antiperovskite halides for *B* = *X* (*X* = Cl, Br, I). Similar to *MA*^+^ in *MA*Pb*X*_3_, an anisotropic atomic group containing a transition metal at the *A* site may result in certain new novel chemical/physical properties, *e.g*., band gap tuning or spin polarization. In this work, three new antiperovskite chalco-halides *M*_3_*AB* (Ba_3_(FeS_4_)Cl, Ba_3_(FeS_4_)Br, and Ba_3_(FeSe_4_)Br), are synthesized. All these chalco-halides showed semiconducting behavior in UV-vis absorption and electrical transport measurements. Meanwhile, an unexpected anti-ferromagnetic phase transition around 100 K was observed during the magnetic susceptibility measurements. The transition is attributed to the antiferromagnetic super-super exchange of Fe^3+^ spins which have the shortest distance of more than 6.267 (2) Å.

## Results and Discussion

**Figure 1:**
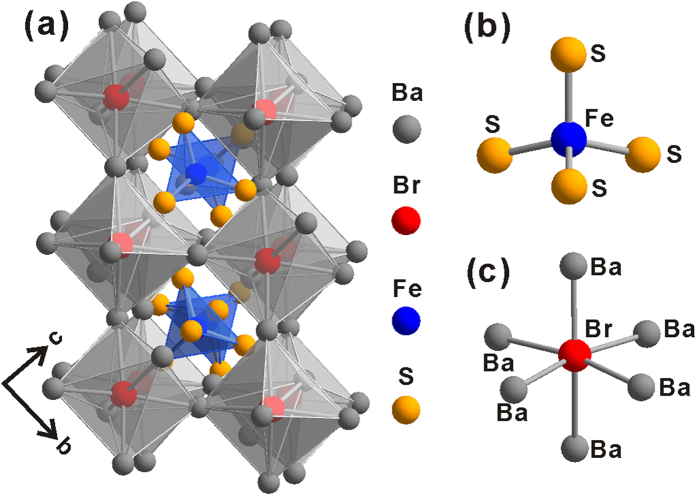
(**a**) Schematic diagram of the crystal structure of Ba_3_(FeS_4_)Br viewing down the *a* axis. (**b**) Coordination environments of (**b**) Fe and (**c**) Br atoms.

Three isostructural compounds (Ba_3_(FeS_4_)Cl, Ba_3_(FeS_4_)Br, Ba_3_(FeSe_4_)Br) are crystallized in the orthorhombic space group *Pnma*, and their crystal structure is shown in Fig. 1a. The structure of Ba_3_(FeS_4_)Br contains two independent Ba sites, one independent Fe site, three independent S sites, and one independent Br site. The structure (*M*_3_*AB*) can be considered as an antiperovskite-like structure, which consists of a 3D octahedral framework of [Ba_3_Br] filled by *A* = FeS_4_ tetrahedra (Fig. 1b). The Br atom at the *B* site is coordinated to six Ba atoms (*M* sites), as shown in Fig. 1c, with an average Br-Ba distance of 3.41 (1) Å, comparable to the Br-Ba distance of 3.42 Å in BaBr_2_. There are two zigzag arrangements for the FeS_4_ tetrahedra: one along the *a* axis (Supplementary Fig. S1a) separated by three Ba atoms with the nearest Fe···Fe distance of 6.267(2) Å and S···S distance of 4.069(3) Å; the other along the *b* axis (Supplementary Fig. S1b) only separated by two Ba atoms with the nearest Fe···Fe and S···S distances of 6.324(1) Å and 3.817(2) Å, respectively. The FeS_4_ tetrahedra (*A* sites) have an average Fe−S distance of 2.246(2) Å. Tolerance factors (*τ*) of the new antiperovskites were calculated as 0.882, 0.843, and 0.872 for Ba_3_(FeS_4_)Cl, Ba_3_(FeS_4_)Br, and Ba_3_(FeSe_4_)Br, respectively.

The oxidation states of these antiperovskites Ba_3_(FeS_4_)Cl, Ba_3_(FeS_4_)Br, and Ba_3_(FeSe_4_)Br can be assigned as Ba^2+^, Fe*Q*_4_^5−^ (Fe^3+^, S^2−^ and Se^2−^), Cl^−^, and Br^−^. Surprisingly, the charge of the *A*-site group (Fe*Q*_4_^5−^) is −5, compared with those in perovskites (+1 for alkali metal or *MA* group, +2 for alkaline-earth metal, +3 for rare earth metal, +4 for Th) and those in antiperovskites (−2 in Li_3_OCl, −3 in Mg_3_SbN, −4 of SiO_4_^4−^ in naturally formed antiperovskite materials Ca_3_(SiO_4_)O). The atomic groups at the *A* site in *ABX*_3_ or *M*_3_*AB* have been found to be *MA*^+^ in *MA*Pb*X*_3_, and SiO_4_^4-^ in Ca_3_(SiO_4_)O. Compared with one single atom at the *A* site, these atomic groups are more anisotropic to cause charge polarization, *e.g.*, dipole moment as that occurring in *MA*Pb*X*_3_^35^. The three compounds Ba_3_(FeS_4_)Cl, Ba_3_(FeS_4_)Br, and Ba_3_(FeSe_4_)Br can be called as a chalco-halides. Note that, five other reported compounds Ba_3_*MQ*_4_*X* (*M* = Ga, In; *Q* = S, Se; *X* = Cl, Br) can also be included in this chalco-halide family, although they have not been noticed to be antiperovskites. Furthermore, if the Ba atoms in these chalco-halides are replaced by Pb, the carrier mobility can greatly be enhanced in the perovskite-like framework of Pb_3_*X* filled by the *A*-site tetrahedral groups, which may be another promising photovoltaic material after *MA*Pb*X*_3_ for the next generation solar cells.

**Figure 2:**
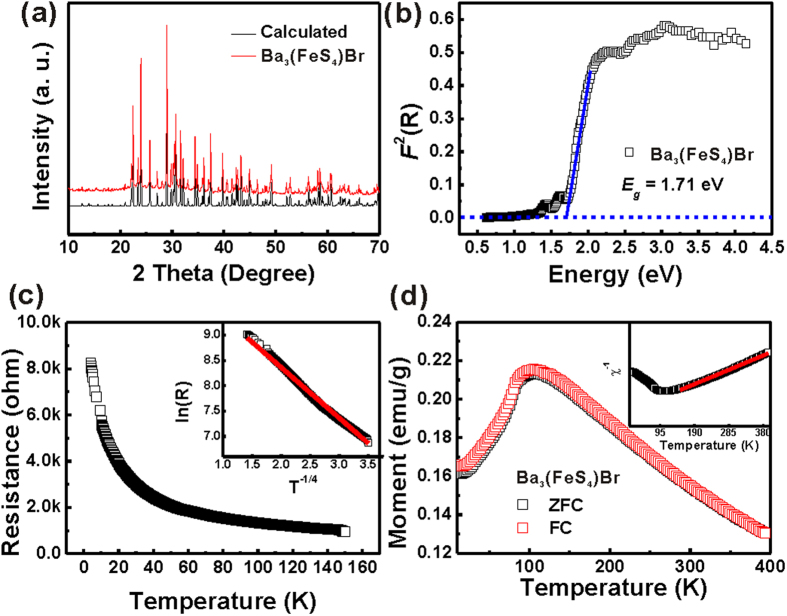
(**a**) Powder X-ray diffraction patterns of Ba_3_(FeS_4_)Br. (**b**) Solid state UV-Vis spectrum of Ba_3_(FeS_4_)Br. (**c**) Temperature dependence of the resistance of Ba_3_(FeS_4_)Br. Inset: *ln(R) vs T*^*−1/4*^ plot of the VRH model. (**d**) Temperature dependence of the magnetization of Ba_3_(FeS_4_)Br. Inset: The inverse magnetic susceptibility *vs* temperature plot. The red line is the linear fit of the magnetic susceptibility data from 400 K to 150 K.

The phase purity of the given powder was indexed on PXRD patterns (Supplementary Fig. S2a for Ba_3_(FeS_4_)Cl and Fig. 2a for Ba_3_(FeS_4_)Br). No extra peaks were observed. Optical properties of the pure Ba_3_(FeS_4_)Cl (Supplementary Fig. S2b) and Ba_3_(FeS_4_)Br (Fig. 2b) samples were investigated by UV-visible diffuse reflectance spectroscopy (UV-Vis). The compounds show sharp absorption edges, indicating semiconductive nature with a rather high absorption coefficient. Consistent with the dark red color of the fine powders, the final band gap energies obtained using the extrapolation method are 1.65 and 1.71 eV from the main absorption edges of Ba_3_(FeS_4_)Cl and Ba_3_(FeS_4_)Br, respectively. The band gaps, which are close to the optimized band gap (1.0−1.8 eV) for solar cells (*MA*PbI_3_, 1.55 eV), are even smaller than that of *MA*PbBr_3_ (2.3 eV)^36^.

Typical exponential temperature dependence was observed in the resistivity of the sample disk, indicating semiconducting behavior (Fig. 2c). There are two well-known models that have been used to describe the semiconducting transport: the small polaron hopping (SPH) model and the variable range hopping (VRH) model. In the SPH model, *ρ(T )* is expressed as *ρ(T )/T µ exp(E*_*P*_*/k*_*B*_*T )*, where the *Ep* is the activation energy^37^; while in the VRH model, *ρ(T )* is expressed as *ρ(T) µ exp(T*_*0*_*/T )*^*1/4*^,where the *T*_*0*_ is the characteristic temperature^38^. Apparently, the resistivity *ρ(T )* of Ba_3_(FeS_4_)Br can be fitted by the VRH model (Fig. 2c inset) instead of the SPH model (Supplementary Fig. S3).

Temperature dependent *DC* magnetic susceptibility of Ba_3_(FeS_4_)Br single crystals is shown in Fig. 2d. It exhibits a broad maximum peak around 100 K and a sharp decrease at 84 K, indicating the presence of antiferromagnetic phase transition below 84 K. Similar transitions, with higher transition temperature of 95 K, are also found in the Ba_3_(FeS_4_)Cl single crystals (Supplementary Fig. S2c). Note that the antiferromagnetic phase transition temperature is much higher than that of Ba_2_BiFeS_5_ (*T*_*N*_ = 35 K)^39^, Ba_2_SbFeS_5_ (*T*_*N*_ = 13 K)^39^, and Ba_3_FeS_5_ (paramagnetic)^40^,^41^. The inverse magnetic susceptibility of Ba_3_(FeS_4_)Br (Fig. 2d inset) shows a typical Curie-Weiss behavior at high temperature. The effective magnetic moment per Fe atom in Ba_3_(FeS_4_)Br is derived to be 2.92 *μ*_*B*_, indicative of the high spin state of the Fe atoms. Linear-dependency of the *M vs H* curves at 50 K, 100 K and 300 K of Ba_3_(FeS_4_)Cl (Supplementary Fig. S2d) and Ba_3_(FeS_4_)Br (Supplementary Fig. S4) also reveals the antiferromagnetic character. It is obvious that the further the magnetic ions separate, the weaker that the spin-spin coupling will be. However, from the crystal structure the nearest Fe-Fe distance in Ba_3_(FeS_4_)Br is 6.267(2) Å, which is too far for the direct spin-spin interaction between Fe atoms. Therefore, it will be of great interest to investigate the origin of the antiferromagnetic order at 84 K.

**Figure 3:**
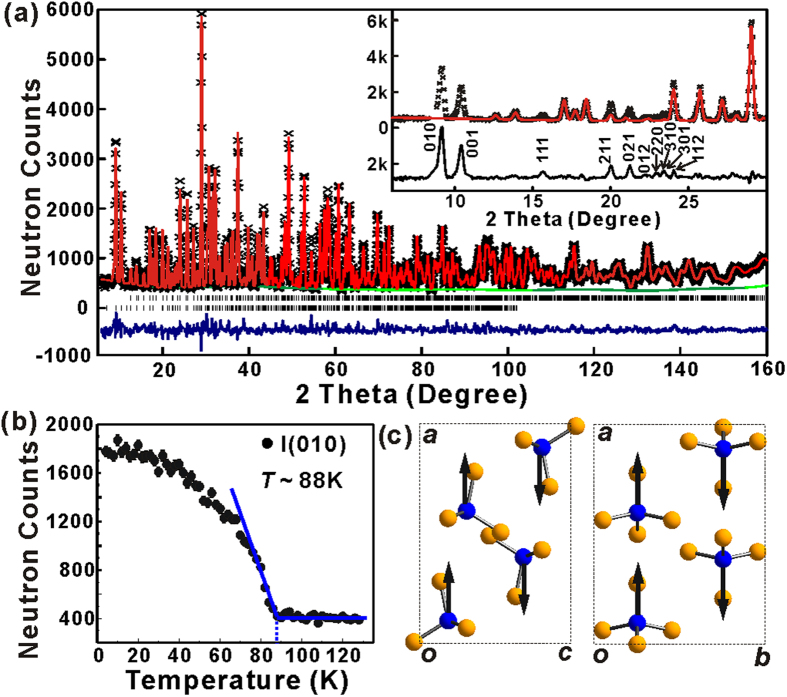
(**a**) Experimental (crosses), calculated (line), and difference (noisy line below observed and calculated patterns) NPD profiles for Ba_3_(FeS_4_)Br at 4 K. Vertical bars indicate the calculated positions of Bragg peaks from the nuclear phase and from the magnetic phase (from the top). *λ* = 1.5398 Å. *R*_wp_ = 0.0558, *R*_p_ = 0.0450, *χ*^2^ = 1.742. Inset: Refinement with nuclear phase only. Some extra peaks from unknown impurities were excluded. (**b**) Temperature dependent intensities of the (010) magnetic reflection. (**c**) Magnetic structure of Ba_3_(FeS_4_)Br view down *b* axis (left) and *c* axis (right). Ba and Br atoms were omitted for clarity.

First, neutron powder diffractions (NPD) experiments were conducted at different temperatures to determine the magnetic structure of Ba_3_(FeS_4_)Br. The refined structural parameters for Ba_3_(FeS_4_)Br at different temperatures are summarized in Supplementary Table S1–S4. At high temperature (130 K and 300 K), all the diffraction peaks of Ba_3_(FeS_4_)Br can be accounted for the orthorhombic crystal structure with *Pnma* space group (Supplementary Fig. S5, S6 and Supplementary Table S1, S2). The NPD pattern measured at 4 K (Fig. 3a) shows clear extra magnetic reflections (difference profile in Fig. 3a inset), which can be indexed in the *Pn’m’a’* Shubnikov group. Temperature dependent intensities of the (010) magnetic reflection (Fig. 3b) imply a magnetic phase transition at ~88 K, which is consistent with the anti-ferromagnetic ordering at 84 K observed in the magnetic susceptibility measurements. The final magnetic structure, with Fe moment of 3.85(3) *μ*_*B*_ along the *a*-axis, is shown in Fig. 3c. Therefore, from the magnetic structure it seems that the low temperature anti-ferromagnetic order originates from the high spin Fe atoms. Next, it is necessary to reveal the spin-spin coupling type in these new antiperovskite chalco-halides.

**Figure 4:**
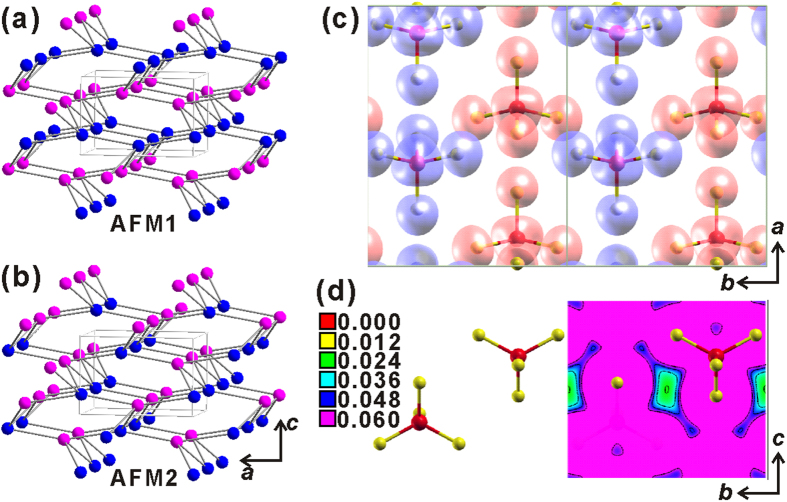
Proposed antiferromagnetic orders (**a**) AFM1 and (**b**) AFM2 with 2 spins up and 2 spins down in first-principles electronic structure calculations. (**c**) Spin density plot of Ba_3_(FeS_4_)Br in the AFM1 state. (**d**) Charge density plane of FeS_4_^5–^ tetrahedra along the *b* axis. For clarity, the Ba and Br atoms have not been shown.

We then performed the spin-polarized first-principles electronic structure calculations on Ba_3_(FeS_4_)Br to understand the electronic and magnetic structures of these new compounds (for computational details, refer to Methods). We considered six possible magnetic orders in a primitive cell with four Fe atoms (spin patterns shown in Supplementary Fig. S7). Their respective energies in the fully relaxed crystal structures with respect to the nonmagnetic state are listed in Supplementary Table S5, which indicate that the AFM1 (Fig. 4a) and AFM2 (Fig. 4b) orders are the ground states with degenerate energy. The common feature of these two magnetic orders is that they both contain antiferromagnetic Fe chains along the *b* axis. Particularly, the spin pattern of the AFM1 order is consistent with the one resolved from the NPD experiment (Fig. 3c).

We further checked the detailed electronic structures of Ba_3_(FeS_4_)Br. The partial density of states (PDOS, Supplementary Fig. S8) reveals that both Fe and S atoms are spin polarized and there are intense hybridizations between the Fe *d* orbitals and the S *p* orbitals. Inspection of the local magnetic moments gives 3.41 *μ*_*B*_ on the Fe atom and 0.14 ~ 0.15 *μ*_*B*_ on the S atom, respectively. The spatial spin density distribution (Fig. 4c) indicates that the Fe and S atoms in the same FeS_4_^5–^ tetrahedron have the same spin polarization orientation. Thus a pair of the nearest Fe atoms can couple with each other via the S atoms in the neighboring FeS_4_^5–^ tetrahedra, albeit the significantly large nearest Fe-Fe distance (>6.2 Å). The calculated (experimental) distances between the nearest S-S atoms in neighboring FeS_4_^5–^ tetrahedra are 4.139 (4.069) Å along the *a* axis and 3.843 (3.817) Å along the *b* axis, respectively. The shorter S-S separation along the *b* axis makes the interaction along this direction stronger than that along the *a* axis, as evidenced by the different charge density intensities between the two neighboring FeS_4_^5–^ tetrahedra depicted in Fig. 4d and Supplementary Fig. S9, respectively. Moreover, along the *b* axis, the four nearest S atoms in the neighboring FeS_4_^5–^ tetrahedra form an approximate rectangle (Fig. 4d and Supplementary Fig. S1b), with two same-spin S atoms in one FeS_4_^5–^ tetrahedron and two same-spin S atoms in the other. Their interactions via the Ba 6*s* orbitals (Supplementary Fig. S1b) prefer an antiferromagnetic coupling, rendering an exchange *J*_*b*_ ~ 29 meV/*S*^2^ with *S* = 3.41 *μ*_*B*_. In contrast, along the *a* axis, three same-spin S atoms in one FeS_4_^5–^ tetrahedron interact with one S atom in the neighboring FeS_4_^5–^ tetrahedron, thus the four S atoms between two neighboring FeS_4_^5–^ tetrahedra also form an S_4_ tetrahedron (Supplementary Fig. S1a). The geometrical frustration as well as the larger S-S distance along the *a* axis do not favor static magnetic order (*J*_*a* _~ 0). This is also the reason that we get the AFM1 and AFM2 orders with degenerate energy (Fig. 4 and Supplementary Table S5). Therefore, the spin-spin coupling between the two nearest Fe atoms is realized by the Fe-S···S-Fe antiferromagnetic super-super exchange along the *b* axis.

In summary, we have synthesized three new chalco-halides Ba_3_(FeS_4_)*X* (*X* = Cl, Br) and Ba_3_(FeSe_4_)Br, all of which all belong to a new type of antiperovskites. The band gap estimations revealed that these antiperovskites are good solar light absorbers. Meanwhile, antiferromagnetic transitions near 100 K were observed in Ba_3_(FeS_4_)*X* (*X* = Cl, Br). The corresponding magnetic structures were resolved by neutron diffractions as well as studied by the first-principles electronic structure calculations. The calculations further showed that the antiferromagnetic ground states feature the opposite spin orientations in Fe···Fe zigzag chains along the *b* axis, and the spin-spin coupling between a pair of the nearest Fe atoms is realized by the Fe−S···S−Fe antiferromagnetic super-super exchange along the *b* axis. These antiperovskite chalco-halides may provide us new ways to searching new promising photovoltaic materials after *MA*Pb*X*_3_ for the next generation solar cells and new physical functions.

## Methods

### Synthesis of Ba_3_(FeS_4_)*X* (*X* = Cl, Br) and Ba_3_(FeSe_4_)Br Single Crystals

All operations were carried out in an Ar-protected glove box. Single crystal samples were synthesized by traditional melting salt method. A mixture of starting materials of Ba pieces (5.00 mmol), Ba*X*_2_ powder (1.00 mmol), Fe powder (2.00 mmol), *Q* (S or Se) powder (8.00 mmol) and KI powder (50 mmol) was loaded in a carbon-coated fused silica tube. The tube was flame-sealed under vacuum (10^−3^ mbar) and heated slowly to 1073 K with a programmable furnace. The reaction was kept at this temperature for 2 days followed by cooling to 773 K at a rate of 2 K/h. Finally, the silica tube was quenched in air. The direct combination reaction at the presence of excess KI flux gave solidified melts. The melts were washed and sonicated by distilled water and dried with acetone. Then the black Ba_3_(FeS_4_)*X* (*X* = Cl, Br) and Ba_3_(FeSe_4_)Br crystals were obtained. The presence of Ba, *X*, Fe and *Q* was confirmed by semi-quantitative energy dispersive X-ray analysis (Supplementary Fig. S10). A number of different crystals were chosen and their average atomic rates of Ba/*X*/Fe/*Q* are summarized in Supplementary Table S6.

### Single Crystal X-ray Crystallography

A single crystal suitable for X-ray diffraction was chosen from the mixture growth *via* the melting salt method. Data collection was performed on a diffractometer equipped with mirror-monochromated Mo-*K*_*α*_ radiation. The structure was solved by direct methods and refined by full-matrix least-squares on *F*^*2*^ using the SHELXTL program package^42^. Multi-scan absorption corrections were performed. The crystal data and refinement details are summarized in Supplementary Table S7.

### Characterization

X-ray diffraction (XRD) patterns were collected on an X-ray diffractometer equipped with a monochromatized source of Cu *K*_*α*_ radiation (λ = 0.15406 nm) at 1.6 kW (40 kV, 40 mA). The patterns were recorded in a slow-scanning mode with 2θ from 10° to 80° with a scan-rate of 1°/min. Simulated patterns were generated using the CIF of the refined structure. Optical diffuse-reflectance measurements were carried out using a spectrophotometer operating from 1800 nm to 300 nm at room temperature. BaSO_4_ powder was used as a 100% reflectance standard. Crystalline samples were ground and spread on a compacted base of BaSO_4_ powder. The reflectance data were converted to absorbance data using the Kubelka-Munk equation to measure the band gap^43^.

### Physical Properties Measurements

Temperature variation of the resistance, *R(T*), was measured using the standard two-probe technique in the Resistivity model collected on a Physical Properties Measurement System (PPMS). For the electric properties measurements, single crystals were ground and pressed into disks, followed by calcination at 773 K for 5 h. Silver paste was applied which act as the contact electrode. Magnetic properties were studied using the PPMS. Temperature-dependent direct-current (*DC*) magnetic susceptibility (*M*–*T*) curve of the sample was measured from 400 to 2 K at 10000 Oe magnetic field under zero field cooling (ZFC) and field cooling (FC) conditions.

### Neutron Diffraction

Neutron powder diffraction (NPD) data ranging from 4 K to 295 K were collected at the NIST Center for Neutron Research (NCNR) using the BT-1 high-resolution neutron powder diffractometer with a Cu(311) monochromator at *λ* = 1.5398 Å. Rietveld structural refinements were performed using GSAS package^44^.

### Electronic Structure Calculation

First-principles calculations were carried out with the Vienna *Ab initio* Simulation Package (VASP)^45^,^46^,^47^, which makes use of the projector augmented wavemethod^48^,^49^. The generalized gradient approximation (GGA) of the Perdew-Burke-Ernzerh^50^ type for the exchange-correlation potential was adopted. The kinetic energy cutoff of the plane-wave basis was chosen to be 350 eV. A 4 × 4 × 6 k-point mesh for the Brillouin zone sampling and the Gaussian smearing technique with a width of 0.05 eV were used. In structure optimization, both cell parameters and internal atomic positions were allowed to relax until the forces were smaller than 0.01 eV/Å. Computational resources have been provided by the Physical Laboratory of High Performance Computing at Renmin University of China. The atomic structure and spin and charge densities were prepared with the XCRYSDEN program^51^.

## Additional Information

**How to cite this article**: Zhang, X. *et al.* Antiperovskite Chalco-Halides Ba_3_(FeS_4_)Cl, Ba_3_(FeS_4_)Br, and Ba_3_(FeSe_4_)Br with Spin Super-Super Exchange. *Sci. Rep.* **5**, 15910; doi: 10.1038/srep15910 (2015).

## Supplementary Material

Supplementary Information
