# Supplementary material for: Antiperovskite Chalco-Halides Ba3(FeS4)Cl, Ba3(FeS4)Br, and Ba3(FeSe4)Br with Spin Super-Super Exchange
Source: Sci Rep. 2015 Nov 3;5:15910. doi: 10.1038/srep15910 (PMC4630630; doi:10.1038/srep15910)
Supplement: Supplementary Information [file srep15910-s1.pdf]

Supporting Information

Antiperovskite Chalco-Halides $Ba_3(FeS_4)Cl$, $Ba_3(FeS_4)Br$ and $Ba_3(FeSe_4)Br$ with Spin Super-Super Exchange

Xian Zhang[1,+], Kai Liu[2,+], Jianqiao He[3], Hui Wu[4,*], Qingzhen Huang[4], Jianhua Lin[1], Zhongyi Lu[2,*], and Fuqiang Huang[1,3,*]

[1]Beijing National Laboratory for Molecular Sciences and State Key Laboratory of Rare Earth Materials Chemistry and Applications, College of Chemistry and Molecular Engineering, Peking University, Beijing 100871, China
[2]Beijing Key Laboratory of Opto-electronic Functional Materials & Micro-nano Devices, Department of Physics, Renmin University of China, Beijing 100872, China
[3]CAS Key Laboratory of Materials for Energy Conversion and State Key Laboratory of High Performance Ceramics and Superfine Microstructure, Shanghai Institute of Ceramics, Chinese Academy of Sciences, Shanghai 200050, China
[4]NIST Center for Neutron Research, National Institute of Standards and Technology, Gaithersburg, MD 20899-6102, USA
* huiwu@nist.gov, zlu@ruc.edu.cn, huangfq@pku.edu.cn
[+]these authors contributed equally to this work

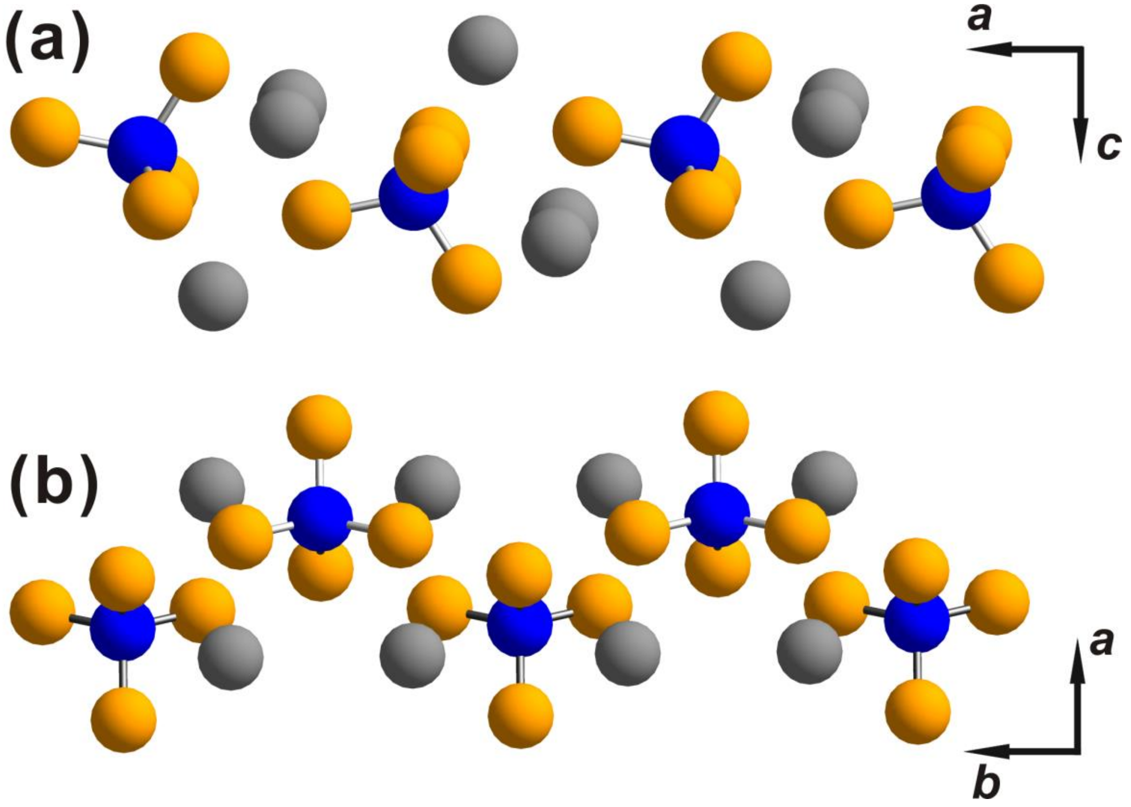

Figure S1. Two type of FeS_4 tetrahedral arrangements along *a* axis (a) and *b* axis (b) in $Ba_3(FeS_4)Br$. Fe, S, and Ba, are represented by blue, orange, and grey spheres, respectively.

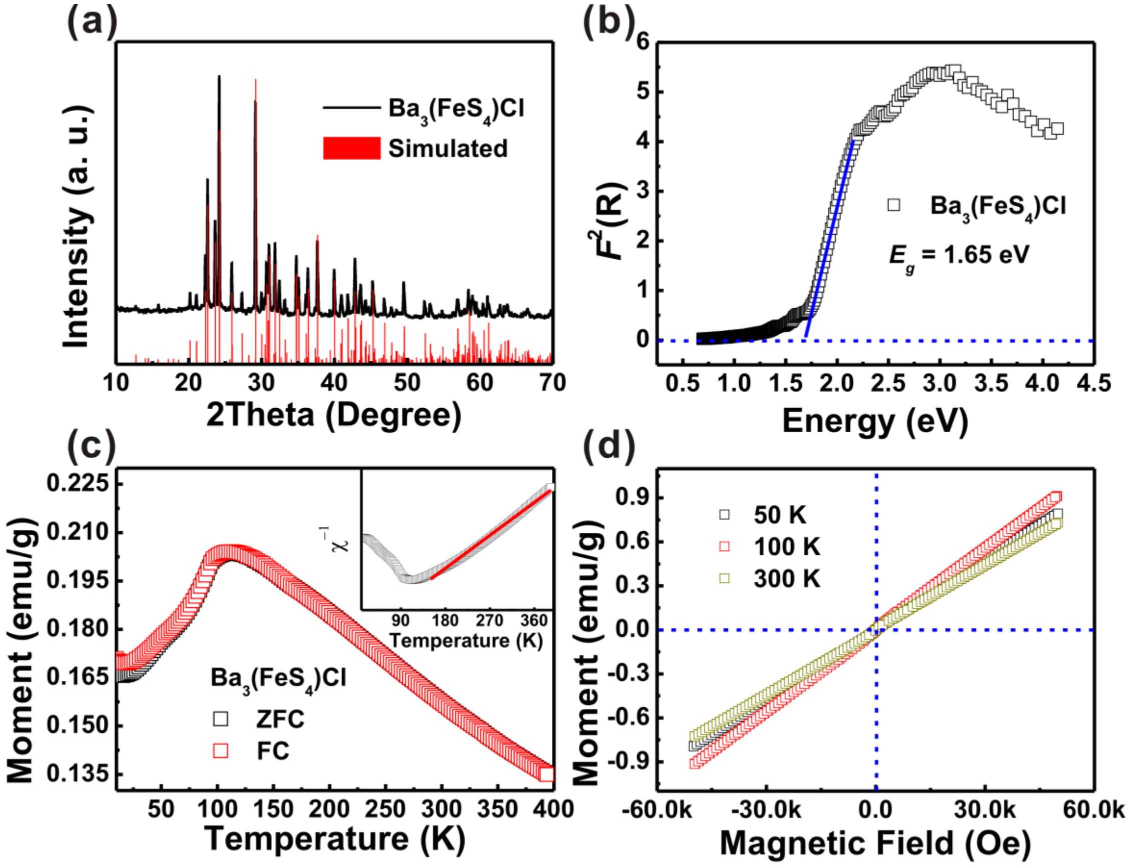

Figure S2. (a) Powder X-ray diffraction patterns of $Ba_3(FeS_4)Cl$. (b) Solid state UV-Vis spectrum of $Ba_3(FeS_4)Cl$. (c) Temperature-dependence of magnetization of $Ba_3(FeS_4)Br$. Inset: The inverse magnetic susceptibility *vs* temperature plot. The red line is the linear fit of the magnetic susceptibility data from 400 K to 150 K. (d) *M vs H* curves of $Ba_3(FeS_4)Cl$ at 50 K, 100 K, and 300 K.

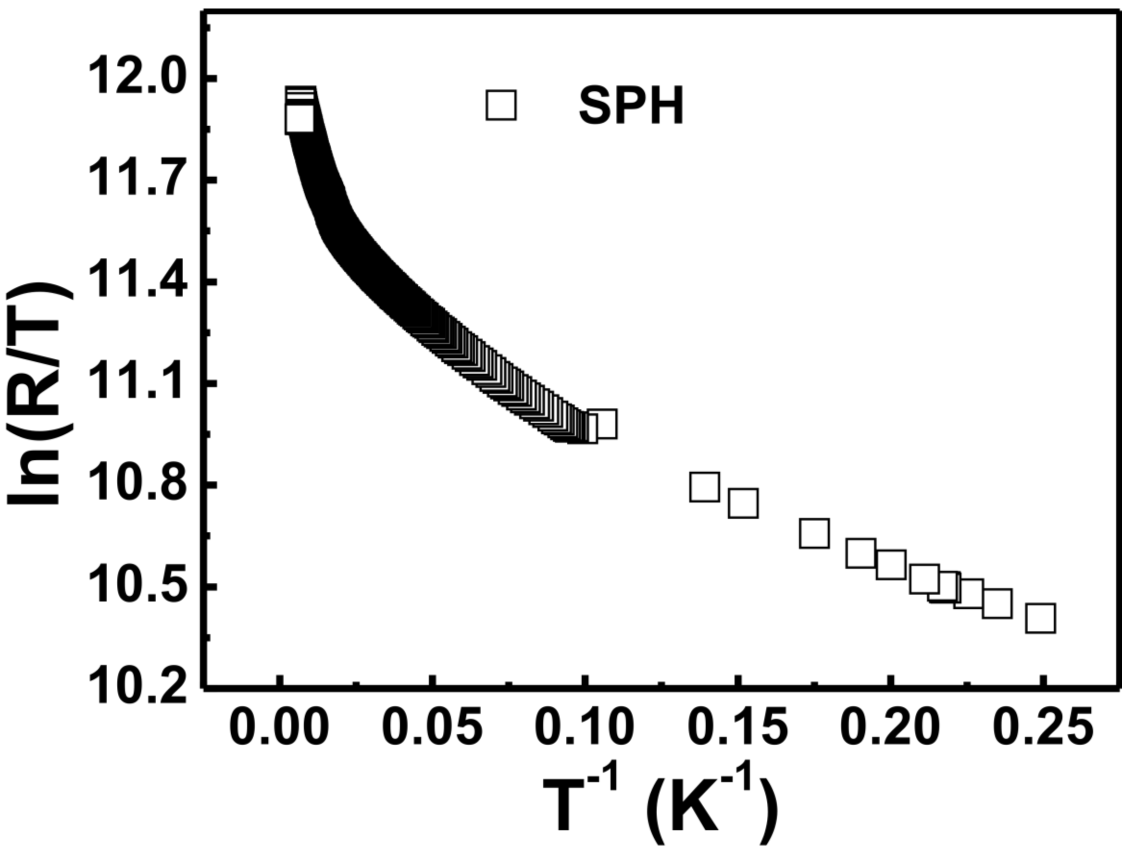

Figure S3. *ln*(R/T) vs T^{-1} plot in the SPH model of $Ba_3(FeS_4)Br$.

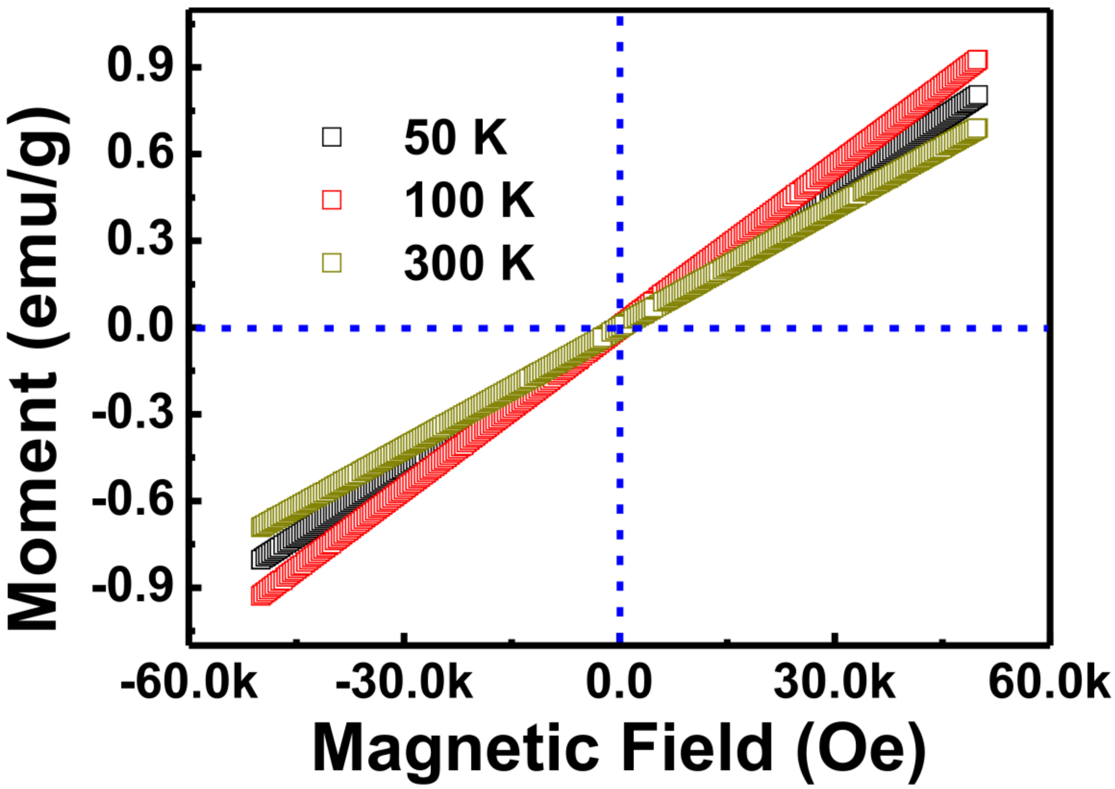

Figure S4. *M vs H* curves of $Ba_3(FeS_4)Br$ at 50 K, 100 K, and 300 K.

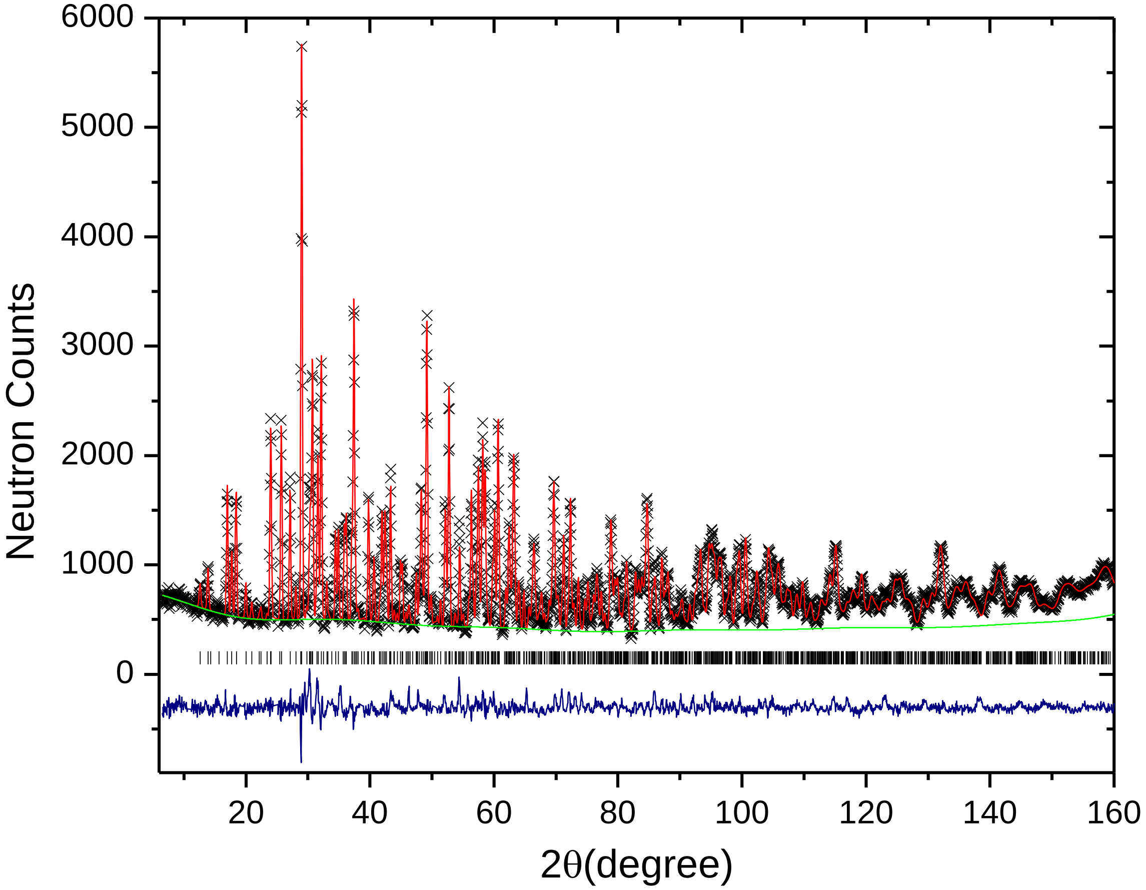

Figure S5. Experimental (circles), calculated (line), and difference (noisy line below observed and calculated patterns) NPD profiles for $Ba_3(FeS_4)Br$ at 130K. Vertical bars indicate the calculated positions of Bragg peaks from the nuclear phase. λ=1.5398Å. R_{wp}=0.0519, R_p=0.0407, χ^2=1.591. Some extra peaks from unknown impurities were excluded.

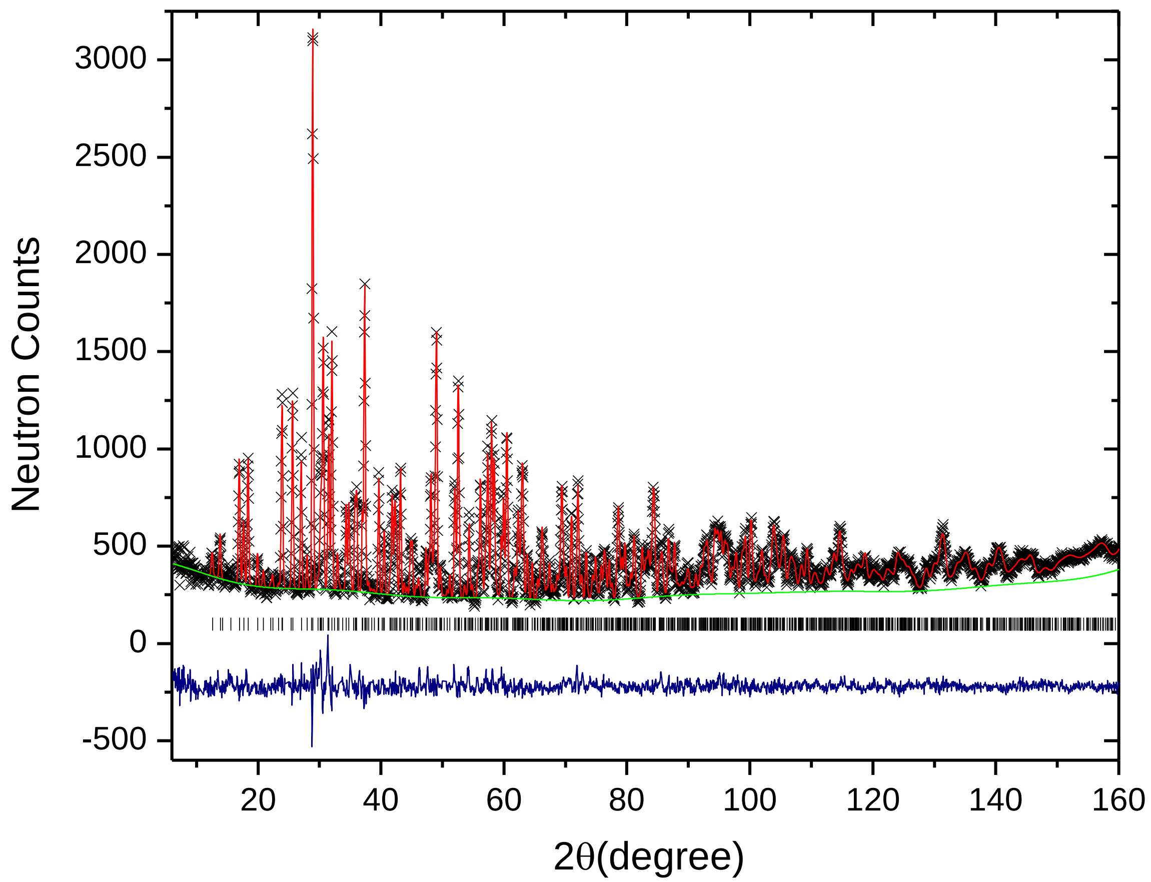

Figure S6. Experimental (circles), calculated (line), and difference (noisy line below observed and calculated patterns) NPD profiles for $Ba_3(FeS_4)Br$ at 295K. Vertical bars indicate the calculated positions of Bragg peaks from the nuclear phase. λ=1.5398Å. R_{wp}=0.0559, R_p=0.0444, χ^2=1.387. Some extra peaks from unknown impurities were excluded.

Table S1. Refined structural parameters of $Ba_3(FeS_4)Br$ at T=4K: Nuclear space group *Pnma*, No. 62, *a* = 12.3430(4) Å, *b* = 9.5901(3) Å, *c* = 8.4468(3) Å, V=999.85(7) $Å^3$; Magnetic symmetry of Shubnikov group: *Pn'm'a'* with Fe moment of 3.85(3) μ_B along *a*-axis direction.

Atom	Site	Occup.	x	y	z	U_{iso} (x100$Å^2$)
Ba	*4c*	1.0	0.4755(3)	0.25	0.9181(5)	0.13(8)
Ba	*8d*	1.0	0.6639(3)	0.9787(2)	0.5884(3)	0.31(5)
Br	*4c*	1.0	0.7326(2)	0.25	0.8301(3)	0.58(7)
Fe	*4c*	1.0	0.6045(1)	0.25	0.3104(3)	0.46(4)
S1	*8d*	1.0	0.5695(3)	0.0484(5)	0.1895(5)	0.40(9)
S2	*4c*	1.0	0.5093(5)	0.25	0.5374(8)	0.8(1)
S3	*4c*	1.0	0.7162(5)	0.75	0.8621(7)	0.5(1)

Table S2. Refined structural parameters of $Ba_3(FeS_4)Br$ at T=130K: Nuclear space group *Pnma*, No. 62, *a* = 12.3636(5) Å, *b* = 9.5974(4) Å, *c* = 8.4596(3) Å, V=1003.7(1) Å^3

Atom	Site	Occup.	x	y	z	U_{iso} (x100Å^2)
Ba	*4c*	1.0	0.4755(3)	0.25	0.9182(5)	0.22(8)
Ba	*8d*	1.0	0.6637(2)	0.9784(2)	0.5880(3)	0.75(5)
Br	*4c*	1.0	0.7325(2)	0.25	0.8302(3)	1.03(7)
Fe	*4c*	1.0	0.6049(1)	0.25	0.3104(3)	0.67(4)
S1	*8d*	1.0	0.5700(4)	0.0481(5)	0.1890(6)	0.79(9)
S2	*4c*	1.0	0.5083(6)	0.25	0.5387(9)	1.2(2)
S3	*4c*	1.0	0.7157(5)	0.75	0.8625(6)	0.7(1)

Table S3. Refined structural parameters of $Ba_3(FeS_4)Br$ at T=295K: Nuclear space group *Pnma*, No. 62, *a* = 12.4081(5) Å, *b* = 9.6132(4) Å, *c* = 8.4859(4) Å, V=1012.2(1) Å^3.

Atom	Site	Occup.	x	y	z	U_{iso} (x100Å^2)
Ba	*4c*	1.0	0.4755(3)	0.25	0.9166(6)	0.8(1)
Ba	*8d*	1.0	0.6619(3)	0.9772(3)	0.5882(4)	1.39(7)
Br	*4c*	1.0	0.7326(3)	0.25	0.8306(4)	1.88(9)
Fe	*4c*	1.0	0.6051(2)	0.25	0.3092(3)	1.11(5)
S1	*8d*	1.0	0.5705(4)	0.0488(6)	0.1881(7)	1.6(1)
S2	*4c*	1.0	0.5100(7)	0.25	0.537(1)	2.0(2)
S3	*4c*	1.0	0.7162(5)	0.75	0.8613(7)	0.6(2)

Table S4. Selected interatomic bond lengths (Å) and angles (º) in Ba_3BrFeS_4

a) T=5K

Bond lengths						Bond angles		
Fe-S1	x2	2.229(4)	Br-Ba1	x1	3.260(4)	S1-Fe-S1		120.31(25)
Fe-S2	x1	2.249(8)	Br-Ba1	x1	3.658(5)	S1-Fe-S2	x2	106.81(14)
Fe-S3	x1	2.256(7)	Br-Ba2	x2	3.4143(31)	S1-Fe-S3	x2	106.21(14)
			Br-Ba2	x2	3.3468(34)	S2-Fe-S3		110.36(26)
Ba1-S1	x2	3.216(6)	Ba2-S1	x1	3.448(5)	Ba1-Br-Ba1		158.21(10)
Ba1-S1	x2	3.054(4)	Ba2-S2	x1	3.255(5)	Ba1-Br-Ba2	x2	83.93(8)
Ba1-S2	x1	3.242(8)	Ba2-S2	x1	3.242(5)	Ba1-Br-Ba2	x2	102.88(9)
Ba1-S3	x1	3.007(8)	Ba2-S3	x1	3.252(5)	Ba1-Br-Ba2	x2	82.01(8)
Ba2-S1	x1	3.627(5)	Ba2-S3	x1	3.552(5)	Ba1-Br-Ba2	x2	93.49(9)
Ba2-S1	x1	3.410(5)				Ba2-Br-Ba2	x2	169.74(9)
						Ba2-Br-Ba2		99.29(11)
						Ba2-Br-Ba2	x2	89.155(31)
						Ba2-Br-Ba2		81.89(10)

b) T=130K

Bond lengths						Bond angles		
Fe-S1	x2	2.235(4)	Br-Ba1	x1	3.264(4)	S1-Fe-S1		120.23(25)
Fe-S2	x1	2.271(8)	Br-Ba1	x1	3.666(5)	S1-Fe-S2	x2	106.83(14)
Fe-S3	x1	2.261(7)	Br-Ba2	x2	3.4228(31)	S1-Fe-S3	x2	106.18(14)
			Br-Ba2	x2	3.3477(34)	S2-Fe-S3		110.47(25)
Ba1-S1	x2	3.220(6)	Ba2-S1	x1	3.461(5)	Ba1-Br-Ba1		158.22(10)
Ba1-S1	x2	3.054(4)	Ba2-S2	x1	3.265(5)	Ba1-Br-Ba2	x2	83.96(8)
Ba1-S2	x1	3.236(8)	Ba2-S2	x1	3.237(5)	Ba1-Br-Ba2	x2	102.97(9)
Ba1-S3	x1	3.005(7)	Ba2-S3	x1	3.257(5)	Ba1-Br-Ba2	x2	81.98(8)
Ba2-S1	x1	3.631(6)	Ba2-S3	x1	3.558(5)	Ba1-Br-Ba2	x2	93.41(9)

Ba2-S1	x1	3.410(5)				Ba2-Br-Ba2 x2	169.66(9)
						Ba2-Br-Ba2	99.23(11)
						Ba2-Br-Ba2 x2	89.225(32)
						Ba2-Br-Ba2	81.79(11)

c) T=295K

Bond lengths						Bond angles	
Fe-S1	x2	2.232(5)	Br-Ba1	x1	3.273(5)	S1-Fe-S1	120.12(31)
Fe-S2	x1	2.267(9)	Br-Ba1	x1	3.672(5)	S1-Fe-S2 x2	107.04(17)
Fe-S3	x1	2.261(8)	Br-Ba2	x2	3.446(4)	S1-Fe-S3 x2	106.20(17)
			Br-Ba2	x2	3.356(4)	S2-Fe-S3	110.07(28)
Ba1-S1	x2	3.231(7)	Ba2-S1	x1	3.462(6)	Ba1-Br-Ba1	158.05(12)
Ba1-S1	x2	3.060(5)	Ba2-S2	x1	3.258(6)	Ba1-Br-Ba2 x2	83.40(10)
Ba1-S2	x1	3.247(10)	Ba2-S2	x1	3.234(7)	Ba1-Br-Ba2 x2	103.58(11)
Ba1-S3	x1	3.034(8)	Ba2-S3	x1	3.255(6)	Ba1-Br-Ba2 x2	82.41(10)
Ba2-S1	x1	3.646(7)	Ba2-S3	x1	3.588(5)	Ba1-Br-Ba2 x2	92.99(11)
Ba2-S1	x1	3.436(6)				Ba2-Br-Ba2 x2	169.50(11)
						Ba2-Br-Ba2	99.10(13)
						Ba2-Br-Ba2 x2	89.58(4)
						Ba2-Br-Ba2	81.21(13)

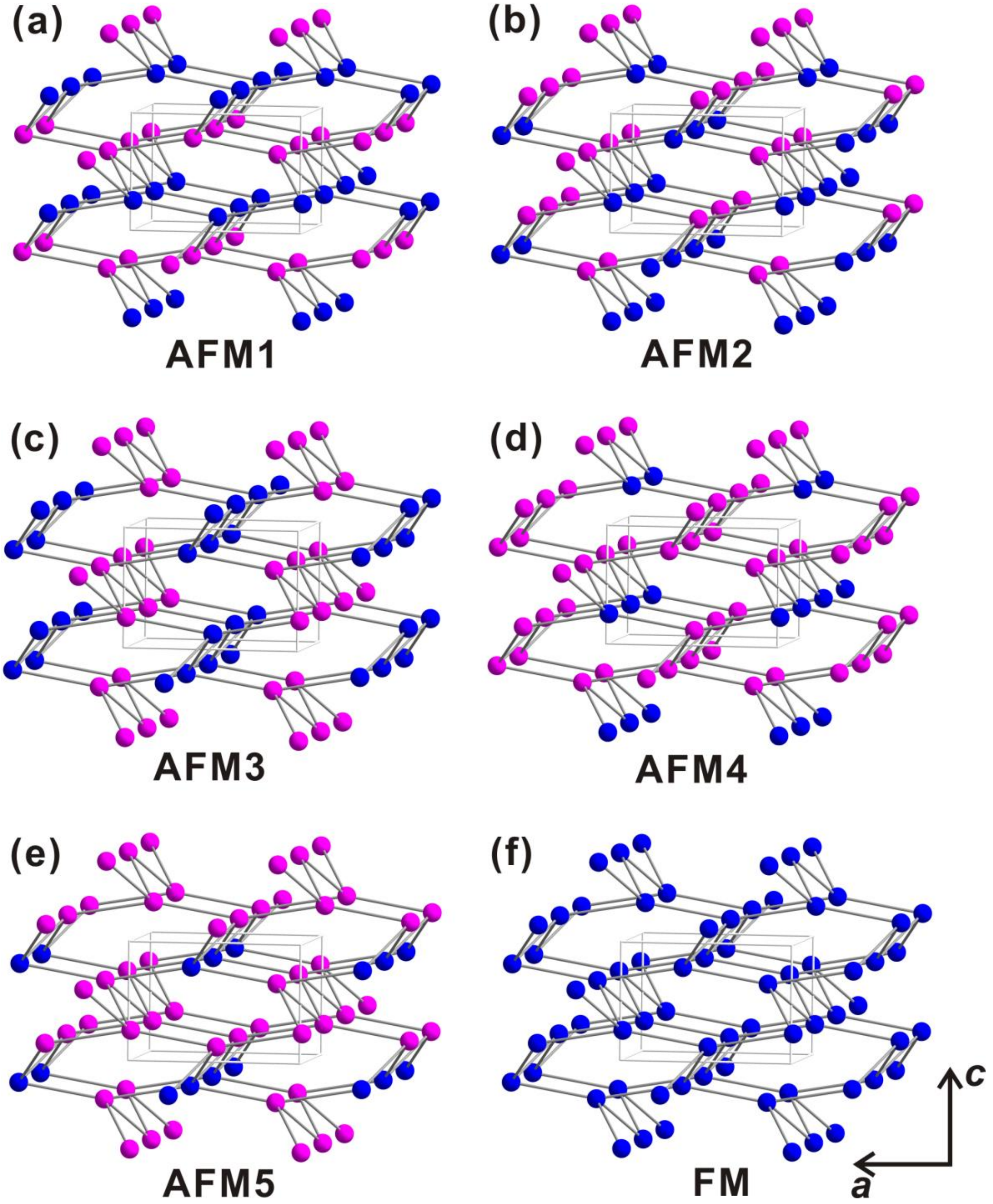

Figure S7. Six possible magnetic orders of $Ba_3(FeS_4)Br$. The red and blue balls denote the Fe atoms with spin up and spin down, respectively. For clarity, the Ba and Br atoms have not been shown.

Table S5. Relative energies of $Ba_3(FeS_4)Br$ in different magnetic states with respect to the nonmagnetic state (unit in eV/Fe).

**Magnetic orders**	***E₀*** **(eV/Fe)**
**AFM1 (2 up and 2 down)**	-1.077
**AFM2 (2 up and 2 down)**	-1.078
**AFM3 (2 up and 2 down)**	-1.019
**AFM4 (3 up and 1 down)**	-1.047
**AFM5 (3 up and 1 down)**	-1.047
**Ferromagnetic (4 spin up)**	-1.015

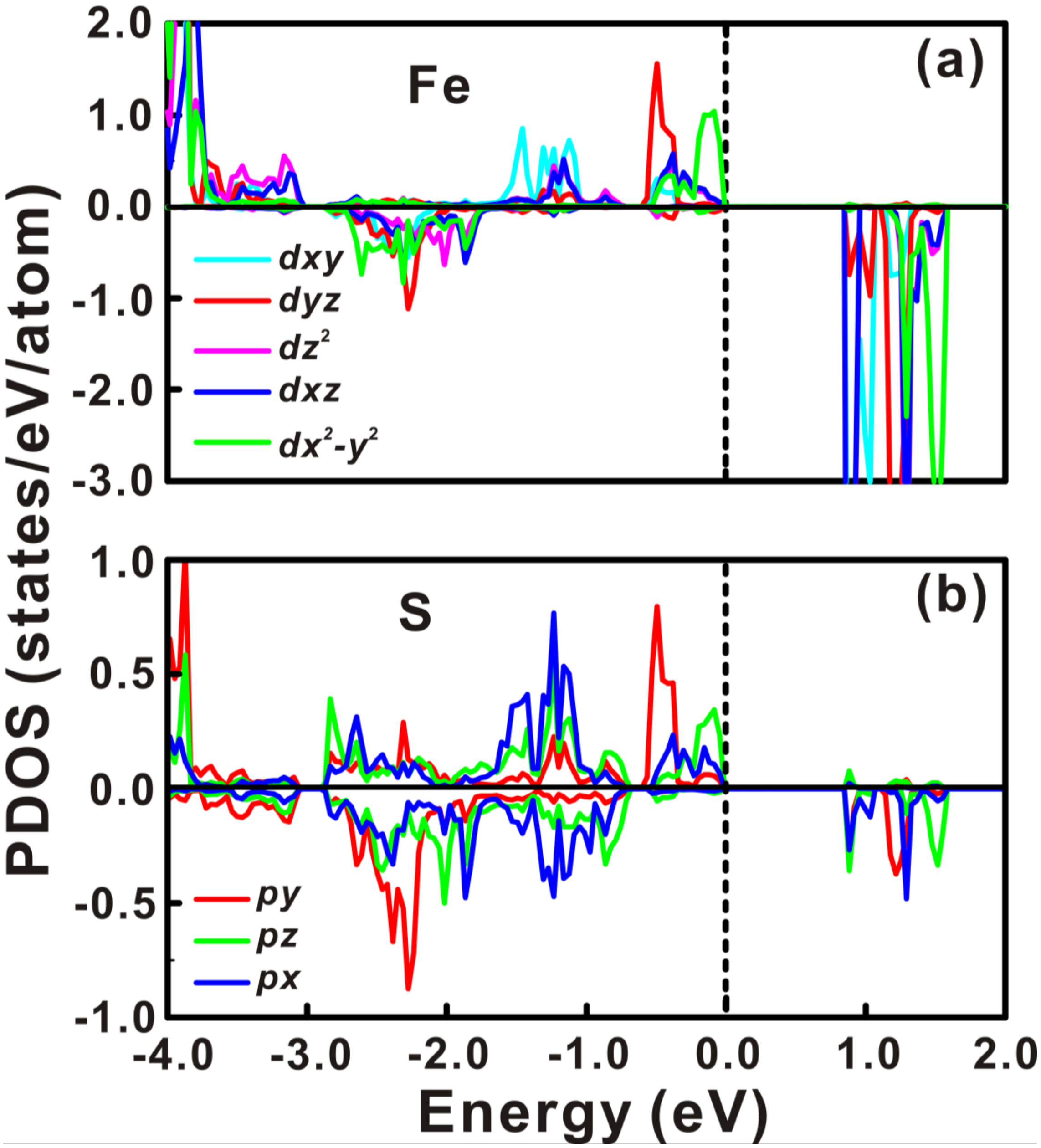

Figure S8. Partial density of states for the Fe and S atoms in the same FeS_4^{5-} tetrahedra of $Ba_3(FeS_4)Br$.

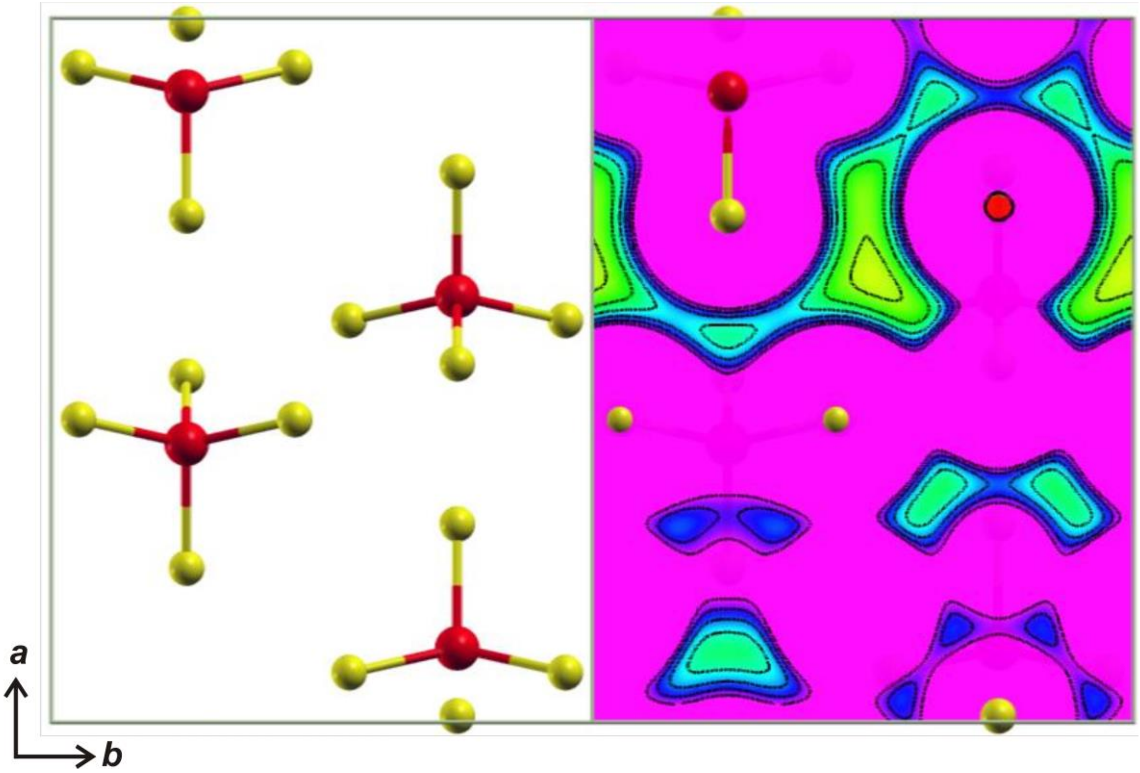

Figure S9. Charge density plane of FeS_4 tetrahedra along *a* axis in $Ba_3(FeS_4)Br$.

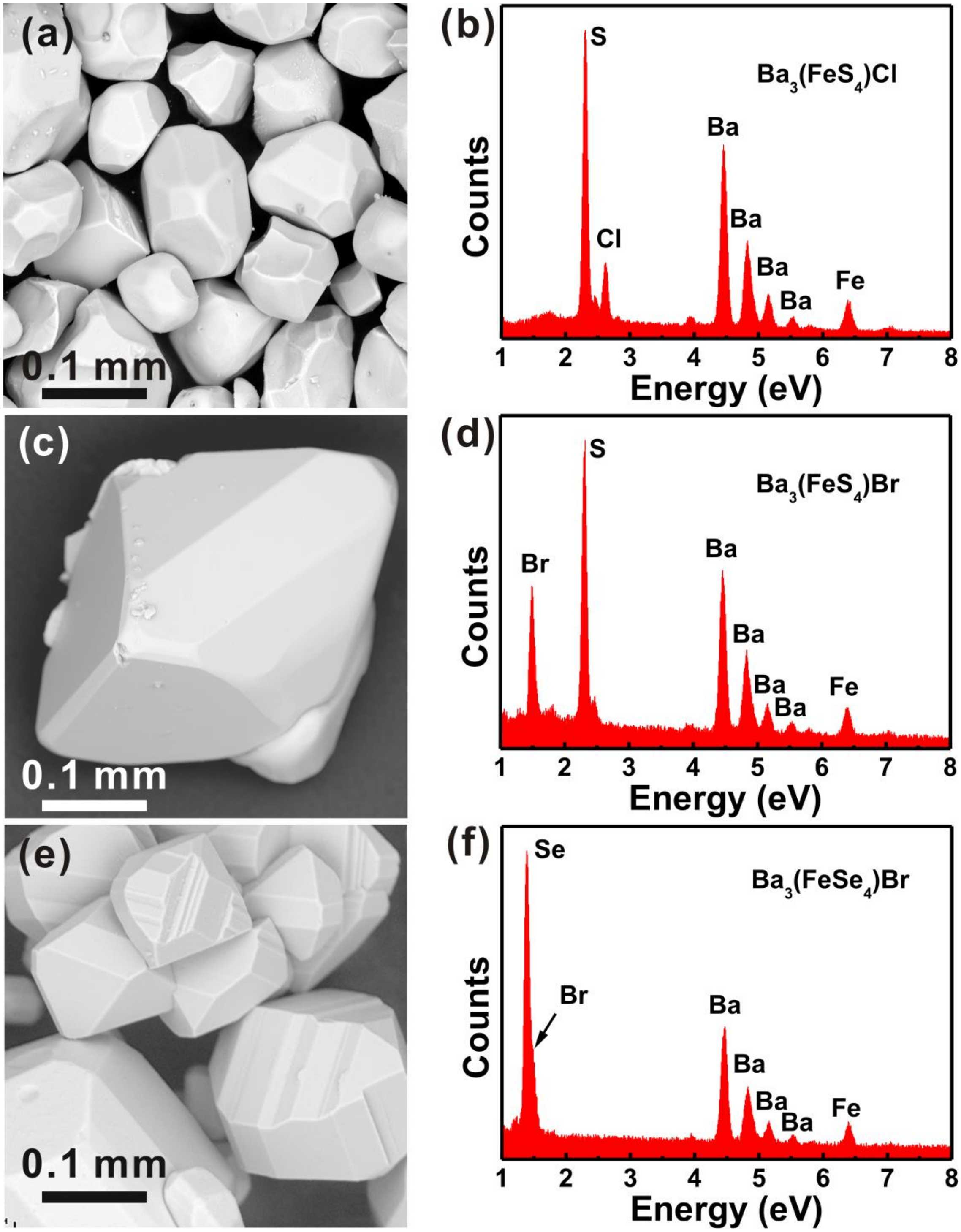

Figure S10. SEM images and EDX spectra of $Ba_3(FeS_4)Cl$ (a, b), $Ba_3(FeS_4)Br$ (c, d), and $Ba_3(FeSe_4)Br$ (e, f) single crystals

Table S6. Average atomic rate of Ba/Fe/*Q*/*X*

Compounds	Atomic rate
**$Ba_3(FeS_4)Cl$**	3.1 : 1.0 : 4.3 : 0.9
**$Ba_3(FeS_4)Br$**	3.1 : 1.0 : 4.1 : 1.1
**$Ba_3(FeSe_4)Br$**	2.9 : 1.0 : 3.9 : 1.2

Table S7. Crystallographic data and details of the structure refinement for $Ba_3(FeS_4)Cl$, $Ba_3(FeS_4)Br$, and $Ba_3(FeSe_4)Br$.

	**1**	**2**	**3**
**empirical formula**	$Ba_3(FeS_4)Cl$	$Ba_3(FeS_4)Br$	$Ba_3(FeSe_4)Br$
**formula weight**	631.5	676.0	863.6
**temperature**	180 K	180 K	180 K
**crystal system**	Orthorhombic	Orthorhombic	Orthorhombic
**space group**	*P*nma	*P*nma	*P*nma
**unit cell dimensions**	a=12.2469(7) Å<br>b=9.5386(5) Å<br>c=8.4231(4) Å	a=12.3618(11)Å<br>b=9.5958(7) Å<br>c=8.4600(6) Å	a=12.7671(6) Å<br>b=9.9039(5) Å<br>c=8.7391(4) Å
**volume**	983.98(9) Å^3	1003.5(1) Å^3	1105.01(9) Å^3
**Z**	4	4	4
**calculated density**	4.263 g/cm^3	4.474 g/cm^3	5.191 g/cm^3
**index ranges**	$-16 \leq h \leq 11$,<br>$-12 \leq k \leq 12$,<br>$-11 \leq l \leq 9$	$-14 \leq h \leq 9$,<br>$-11 \leq k \leq 8$,<br>$-10 \leq l \leq 7$	$-15 \leq h \leq 11$,<br>$-11 \leq k \leq 7$,<br>$-10 \leq l \leq 8$
**unique reflections**	1255	935	1304
**goodness-of-fit on F^2**	1.063	1.016	0.988
**R [I>2σ(I)]**	0.0260	0.0263	0.0201
**wR2 [$I>2\sigma(I)$]**	0.0392	0.0546	0.0341
**R (all)**	0.0387	0.0343	0.0277
**wR2 (all)**	0.0435	0.0579	0.0365
